# Mechanical and Thermal Degradation-Related Performance of Recycled LDPE from Post-Consumer Waste

**DOI:** 10.3390/polym16202863

**Published:** 2024-10-10

**Authors:** Miroslav Müller, Viktor Kolář, Rajesh Kumar Mishra

**Affiliations:** Department of Material Science and Manufacturing Technology, Faculty of Engineering, Czech University of Life Sciences Prague, 165 00 Prague, Czech Republic; muller@tf.czu.cz (M.M.); vkolar@tf.czu.cz (V.K.)

**Keywords:** post-consumer waste, low-density polyethylene, injection molding technology, cyclic fatigue, mechanical properties, recycling, thermal degradation, Fourier transform infrared spectroscopy, differential scanning calorimetry, dynamic mechanical analysis

## Abstract

This paper presents research aimed at laboratory experiments on static and cyclic fatigue testing of low-density polyethylene (LDPE) recovered from post-consumer waste in order to develop a recycled product exhibiting satisfactory mechanical and thermo-mechanical properties. The results of the cyclic fatigue tests set up to 80% of the maximum load in static tensile testing demonstrated satisfactory functionality of the recycled material developed by using the injection molding process. There was no significant change in the tensile strength under static and cyclic fatigue tests. Under cyclic loading, there was a quasi-static effect manifested by plastic deformation, and the displacement increased significantly. The static and cyclic tensile tests indicated improvement in the mechanical performance of the recycled LDPE as compared to the virgin material, owing to the high quality of the regranulates. Fourier Transform Infrared Spectroscopy (FTIR) was conducted to analyze the functional groups in virgin and recycled LDPE samples. The analysis showed no significant change in the transmittance spectra. The thermal degradation performance was also analyzed by Differential Scanning Calorimetry (DSC) and Dynamic Mechanical Analysis (DMA). The results were quite similar for both virgin and recycled LDPE.

## 1. Introduction

The world’s demand for plastic materials continues to grow. As per reports, the amount of plastic materials in circulation will grow from 236 to 417 million tons by 2030 [1]. It is necessary to be aware of the advantages and the environmental hazards associated with the massive use of plastic materials [2]. Global production of plastic materials has increased sharply due to their relatively lower cost, easier production and adequate mechanical performance [3]. The disadvantage lies in ensuring the return of the post-consumer waste material to the production process [4]. Depending on the infrastructure of each country, the treatment of post-consumer plastic waste differs [2]. Post-consumer waste reduction, reuse and recycling are the main approaches to reducing hazardous plastics released into the environment.

In recent years, the concepts of sustainability and circular economy have been linked to reducing the use of natural resources and minimizing emissions [5]. By following these practices, the negative environmental impacts of plastic materials can be significantly reduced.

LDPE is one of the oldest types of polyethylene (PE), characterized by its low density (0.910–0.955 g × cm^3^). When produced using the radical polymerization method, the higher pressure results in a lower crystallization and a higher amount of chain branching. This results in increased flexibility and reduced tensile strength [6,7].

LDPE represents a significant volume of polyolefins found in packaging waste materials (bags, pallet wrap, agricultural films, shopping bags, etc.). These wastes should be reusable [3,8,9,10]. Polyolefins are an important group of thermoplastic packaging materials produced worldwide. The flexible packaging industry relies on LDPE as one of the most commonly used polymers due to its low cost and excellent mechanical properties [10]. The most widely used treatment for LDPE flexible film-based waste material involves cleaning, mechanical crushing and then compaction for feeding into an extruder [10]. LDPE regranulates are characterized by their high quality, resulting from successive processing steps. Firstly, the material is prepared on a sorting line, where the unwanted impurities are excluded. The material is selected for the product, followed by a three-phase washing process. This is characterized by the separation of impurities using friction and flotation technology. The next step is the continuous automatic filtration during the regranulation process itself. The melting of the already washed and dried material is carried out. During this process, the remaining impurities are filtered out and, if necessary, required dyes are added.

LDPE exhibits adequate mechanical properties that are significantly affected by the aging process [8,11]. Moreno et al. [11] point out that the resistance to photochemical aging can be reduced by adding fillers (e.g., pine wood waste) to the recycled LDPE granulates. Adding fillers to recycled plastics is one of the desirable methods to develop new materials that can reduce cost and improve mechanical properties such as strength and modulus [3]. It is considered a material with a high strength-to-density ratio, high chemical resistance and several other advantages [12]. On the other hand, flexible synthetic polymer-based packaging material is highly durable throughout its lifetime and poses a serious environmental problem [13]. When LDPE is recycled again, some physical and mechanical properties may be affected [14,15].

To ensure efficient and safe recycling, contamination of recycled plastics by consumers must be eliminated. This issue was addressed by Núnez et al., who sought to effectively eliminate hazardous contaminants from recycled plastics [9]. Consumer waste-based recycled LDPE contains a large amount of contaminants compared to other types of plastics, e.g., polyethylene terephthalate (PET) [9,16].

Mechanical recycling of consumer waste-based plastics remains the preferred option for environmental and energy reasons [8]. Recycling plastic waste can reduce the ecological footprint resulting from the use of such materials. Ideal recycling in material recovery should produce materials similar to the original or virgin products [17]. Post-consumer waste utilization is undoubtedly part of the solution to the environmental problem associated with the production of polymeric materials [18].

Currently available recycling technologies include mechanical recycling, i.e., direct melt processing in an extrusion line. This is an efficient and effective recycling method. It has a wide range of applications for different types of plastics [19]. The extrusion line uses heat and rotating augers to plasticize, and a homogeneous string is formed at the end of the extrusion process. The extruded polymer is pulverized into the final granulate after cooling. The thermal conductivity and viscous shear that act on the polymeric material during extrusion lead to thermo-oxidation, chain branching or crosslinking [20,21,22]. These processes might result in the deterioration of the mechanical properties of the recycled polymers [20]. LDPE-based packaging exhibits a relatively shorter circulation time, as it is not stabilized against degradation during reprocessing. This might cause problems with the recycling and deterioration of mechanical properties [23]. Pedroso and Rosa [14] reported that the mechanical properties of recycled plastics are inferior compared to the same products made from virgin materials. Bertin and Robin [8], on the other hand, claim that the recycled products show tensile properties close to those of virgin materials.

Recycled polymeric materials are very often considered inferior to those of the original materials [5,24]. Therefore, it is imperative to test recycled plastics in terms of static and dynamic mechanical performance, considering the aspect of cyclic fatigue during their usage.

The fatigue behavior of polymers is influenced by a number of parameters, e.g., the amplitude, intensity and frequency of applied stress [25]. As a result, microcracks can form in polymers. Cracking is characterized as the gradual accumulation of plastic deformation in materials that are subjected to cyclic loading with non-zero mean stress. The accumulation of plastic strain is an important aspect of fatigue damage in materials. The microcracks can ultimately lead to overall degradation of the polymeric materials and affect their long-term durability [25].

Changes in the physical and mechanical properties of recycled plastics have been demonstrated and compared to virgin plastics. Therefore, research activities need to focus on measuring the quality of new regranulate-based recycled polymeric materials [5]. Significant changes also occur due to the addition of fillers to polymers during their production process [26,27]. The recycled materials and products should have reliable mechanical properties throughout their lifetime [28,29].

Improving the knowledge of processing and recycling is an integral part of research activities in the field of polymeric materials. This is a pathway towards a circular economy that takes into account the environmental impacts of recycling such materials [30,31,32].

The aim of current research in the field of material recovery from post-consumer wastes is to describe the mechanical properties from a sustainable development perspective. Further, the assessment of the possibilities for material recycling with respect to mechanical performances is carried out. The research aims to increase the recovery and added value of post-consumer waste-based LDPE. The current research focuses on monitoring the changes in mechanical as well as thermo-mechanical properties considering their fatigue life under cyclic loading.

The research reported in this paper focuses on one of the possible technologies for the production of recycled LDPE based on post-consumer waste by using the injection molding technique. For the production of classic LDPE packaging films, conventional film extrusion and blow molding are more advantageous and therefore commonly used. However, one of the possible practical methods to produce the recycled LDPE material requires injection molding technology. Research on injection-molded recycled LDPE based on post-consumer waste is not significantly explored in this area. From the point of view of low-cycle fatigue tests, no instances in the literature have been reported. This approach was chosen for the verification of the useful mechanical properties of virgin and recycled LDPE based on post-consumer waste. The purity of the recycled materials was also evaluated using FTIR spectroscopy. Thermal degradation behavior was studied using DSC and DMA. The findings are useful to understand the industrial possibilities of manufacturing injection-molded recycled LDPE using post-consumer waste for useful packaging applications.

## 2. Materials and Methods

### 2.1. Materials

Recycled LDPE from ETW Ltd. (ETW s.r.o., Nelahozeves, Czech Republic) was used as the primary material for this research. This was collected in the form of used municipal waste bags. The material supplied is suitable for extrusion and blowing as per the supplier guidelines. According to the material data sheet supplied by the granulate manufacturer, the basic properties of the granulate and recommended processing methods suitable for extrusion and blowing are given as follows: The MFI (melt flow index) was given as 0.5 to 0.7 g/10 min and specific gravity was given as 0.94 g/cm^3^; tensile strength was 15–22 MPa (at 23 °C, and testing speed of 50 mm/min); elongation at break was 550%; breaking strength was approx. 14–20 MPa (at 23 °C, and testing speed of 50 mm/min).

Despite being a material with minimal water absorption capacity, the moisture content in the granulates was found to be approximately 15%. To reduce the moisture content of the granulates, a hot air-drying process was used in a Memmert UN30m+ (Unimed Praha s. r. o., Vestec u Prahy, Czech Republic). The granulate was dried at 40 °C for 24 h with 100% air circulation. The granulate was then removed, and an air humidity value of 8% was measured. After drying, the granulates were used for the production of samples by injection molding.

### 2.2. Methods

#### 2.2.1. Production of Test Specimens

The test specimens were manufactured on a 35 E injection molding machine (PLASTCOMPANY s. r. o., Brno, Czech Republic) with a two-plate clamping system and a maximum clamping force of 350 kN. The injection molding machine was equipped with a double cold injection mold from PLASTCOMPANY s. r. o. with a cavity volume of 26.6 cm^3^ and a weight of 86 kg (mold weight).

The parameters of the injection device setup were:

- Temperatures on the screw: t_1_ = 200 °C; t_2_ = 220 °C; t_3_ = 224 °C; t_4_ = 220 °C; t_5_ = 230 °C
- Clamping force: 303 kN
- Maximum filling pressure: 90 bar

**Figure 1:**
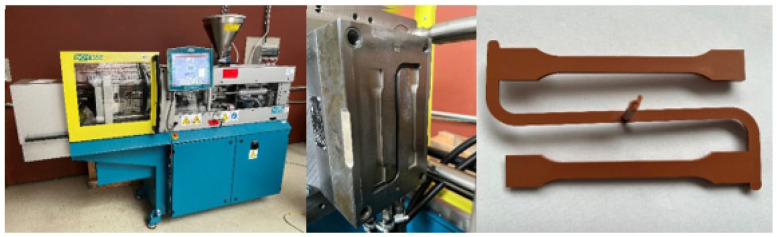
35E injection molding machine with detail of the mold and test specimens produced.

The preparation of the samples is shown in Figure 1.

**Figure 2:**
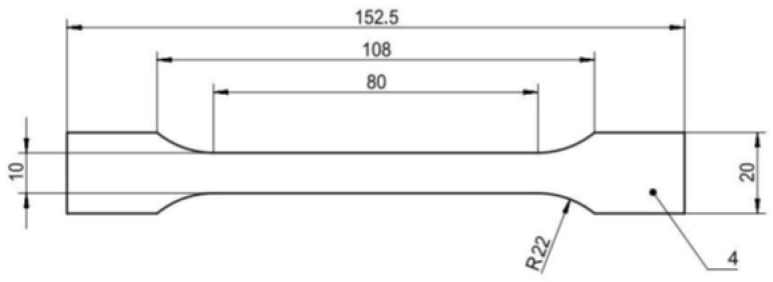
Test specimen defined in standard ČSN EN ISO 527-2 Plastics—Determination of tensile properties—Part 2: Test conditions for molded plastics [33].

The shape and dimension of the test specimens were determined by the cavity of the injection mold, which corresponds to the dimensional regulation defined in standard ČSN EN ISO 527-2 Plastics—Determination of tensile properties—Part 2: Test conditions for molded plastics [33]. A drawing of the test specimen is given in Figure 2. The dimensions are in mm.

#### 2.2.2. Testing of Mechanical Properties

The test specimens were subjected to tensile loading using test equipment LABTest 5.50 ST (LABORTECH s.r.o., Opava, Czech Republic), with a measuring unit AST KAF 50 kN (LABORTECH s.r.o., Opava, Czech Republic) and the evaluation software Test&Motion (version 4.5.0.15, LABORTECH s.r.o., Opava, Czech Republic).

In practice, a large number of polymeric materials are repeatedly stressed and deformed. For this reason, the materials are subjected not only to static but also to cyclic tests, which may have alternating, pulsating or different load patterns [34]. For the research, 6 test specimens were used for each test parameter. The static test was performed at a test speed of 100 mm × min^−1^. The cyclic test was set up as a progressive test, i.e., within one full cycle (1000 loading cycles), and the amplitude of the cyclic stress was changed 4 times (every 250 cycles). The amplitudes were set based on the static test, or the maximum force required to completely break the test specimen. It was found to be 537 ± 21.93 N for recycled LDPE material from post-consumer waste and 414.4 ± 21.6 N for virgin LDPE.

The low cyclic loading of the test specimens consisted of gradual loading. The progressive loading was set from 5% to 20% with a repetition of 250 cycles, followed by a second loading cycle from 5% to 40% (250 cycles), followed by a third loading cycle from 5% to 60% (250 cycles), and a final loading cycle from 5% to 80% (250 cycles). The percentage calculation of the loading force was determined from the reference value of the static tensile test of virgin and recycled LDPE from post-consumer waste. The loading forces are shown in Table 1 and Table 2.

##### Testing of Recycled LDPE from Post-Consumer Waste

The individual amplitudes of the progressive cyclic tests were set according to Table 1. Each variant of the experiment contained 7 test specimens at different test speeds, i.e., the static test at 20, 30, 40 and 50 mm × min^−1^ (marked as “Type of test”).

##### Testing of Virgin LDPE

The individual amplitudes of the progressive cyclic tests were set according to Table 2. Each variant of the experiment contained 7 test specimens at different test speeds, i.e., the static test at 20, 30, 40 and 50 mm × min^−1^ (marked as “Type of test”).

After completion of the 1000^th^ cycle, the cyclic tensile test was followed by a static test with a test speed of 100 mm × min^−1^ until complete failure of the test specimen.

The Δ displacement value represents the viscoelastic behavior of the polymer, i.e., the difference in displacement between the 1^st^ and 1000^th^ cycles.

**Figure 3:**
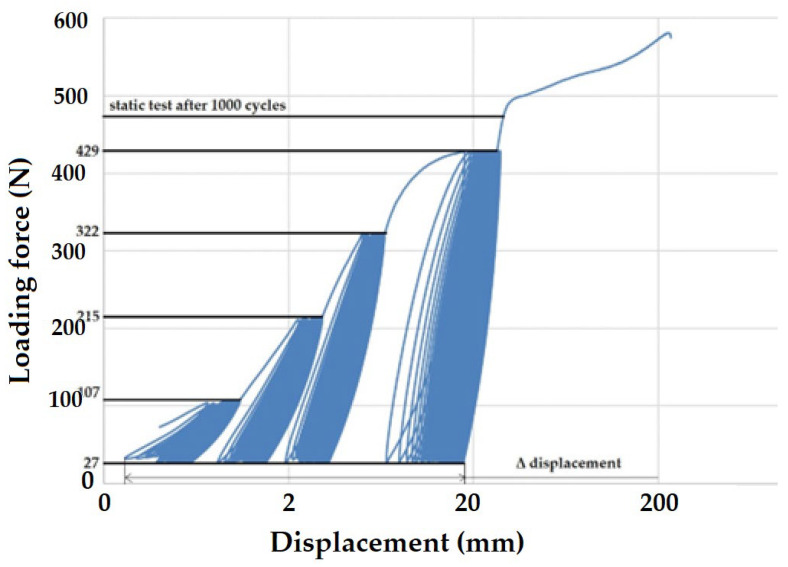
Principle of progressive cyclic test—loading force values for recycled LDPE from post-consumer waste.

Figure 3 shows the principle of progressive cyclic testing and loading force values for recycled LDPE from post-consumer waste.

#### 2.2.3. Statistical Evaluation of Measured Data

Measured values were subjected to statistical analysis using ANOVA F-test using STATISTICA 14 software (version 14.0.0.15, StatSoft CR, Prague, Czech Republic). The significance level of the statistical test was set at 0.05. A statistically significant effect was achieved at a parameter value of *p* < 0.05. A statistically insignificant effect was achieved at a parameter value of *p* > 0.05.

#### 2.2.4. Scanning Electron Microscopy (SEM) Analysis

The fracture surface of the tested LDPE samples formed by injection molding was examined using a MIRA 3 TESCAN device (Tescan Brno s.r.o., Brno, Czech Republic) with an accelerating voltage of 10 kV and an Oxford SE detector (Tescan Brno s.r.o., Brno, Czech Republic). Since this is a non-conductive material, it was necessary to apply a layer of conductive material (gold) before the analysis. The surface of the test samples was gold-plated for SEM analysis with Quorum Q150R ES-Sputtering Deposition Rate (Tescan Brno s.r.o., Brno, Czech Republic).

#### 2.2.5. Fourier Transform Infrared Spectroscopy (FTIR)

FTIR was used to determine the functional groups in the virgin and recycled LDPE from post-consumer waste based on the peak values. The chemical structure of the virgin LDPE and recycled LDPE was confirmed by recording their IR spectra. Attenuated total reflection (ATR) infrared spectroscopy (NicoletiZ10, Thermo Fisher Scientific Corporation, Regensburg, Germany) was used in order to understand the chemical structure of recycled and virgin LDPE samples. Sixteen scans were used for each sample using Zinc Selenide (ZnSe); the spectral range was 4000–500 cm^−1^ with a resolution of 4/cm.

#### 2.2.6. Differential Scanning Calorimetry (DSC)

The melting and crystallization behavior of virgin as well as recycled LDPE from post-consumer waste were investigated using a Perkin Elmer DSC 6 instrument using Pyris, version 2024 software (Mettler Toledo, s.r.o., Prague, Czech Republic). The entire measurement takes place in a nitrogen atmosphere. A sample weighing 10 mg was weighed out for measurement. The sample was heated at a rate of 5 °C per minute from a temperature of 20 °C to a temperature of 200 °C. The crystallinity [%] of the samples was estimated from the enthalpy of LDPE, using the ratio between the heat of fusion of the studied material and the heat of fusion of an infinite crystal of the same material taken from the literature [35,36,37,38]. The crystallinity was measured during the second heating cycle in the DSC procedure.

(1) $$\%Crystalinity=\left(\frac{∆H_{m}}{∆H_{100}}\right)\times 100\%$$

where, ∆*H_m_* is the enthalpy absorbed by the test sample during the heating process and ∆*H*_100_ is the enthalpy absorbed by the sample during the crystallization-melting process. It was taken from the literature for LDPE [35,36,37,38].

#### 2.2.7. Dynamic Mechanical Analysis (DMA)

The dynamic mechanical analysis of the virgin as well as recycled LDPE from post-consumer waste was carried out on a DMA DX04T RMI device (Mettler Toledo, s.r.o., Prague, Czech Republic) under tensile mode. The measurement was performed at a constant frequency of 1 Hz, a strain amplitude of 0.05%, a temperature range of 0 to 100 °C with a heating rate of 5 °C per minute and a 30 mm gap between arms. The samples were prepared by cutting strips 10 mm wide and 40 mm long. A total of 10 samples were measured for the virgin as well as recycled LDPE from post-consumer waste.

## 3. Results and Discussion

### 3.1. Mechanical Properties

Virgin or recycled LDPE from post-consumer waste is applicable in various fields. However, it is subject to real-time cyclic loading. Material integrity may fail during real use. For that reason, it is necessary to understand and have clear data on the fatigue life so that it is possible to reliably apply this material in practical fields [39]. In contrast to metallic materials, fatigue in polymeric materials is more dependent on the speed and frequency of loading, which can have a significantly negative effect on the mechanical properties [39,40,41,42]. Estimation of fatigue life is essential for the application of polymeric materials in various fields [43].

**Figure 4:**
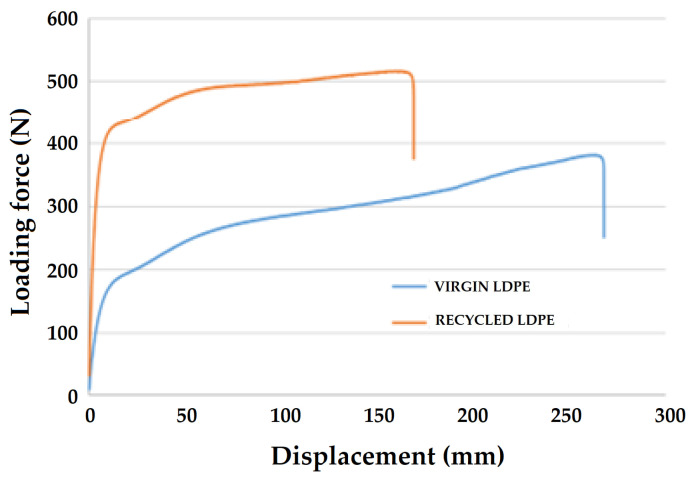
Dependence of loading force on displacement for virgin and recycled LDPE from post-consumer waste.

Figure 4 shows an example force-displacement diagram for virgin and recycled LDPE from post-consumer waste.

The results show a significant difference in the two tested materials during the static tensile test. The yield strengths for virgin and recycled LDPE were 5.23 ± 0.53 MPa and 10.53 ± 0.45 MPa, respectively. The loading force corresponding to the yield strength of virgin LDPE was 209 ± 21 N. It can be observed that some amplitudes of the progressive cyclic tests listed in Table 2 were set above the yield strength of the tested virgin LDPE material. In contrast, the loading force corresponding to the yield strength of recycled LDPE from post-consumer waste was 421 ± 21 N. The amplitude of the sequential cyclic test for one test of recycled LDPE from post-consumer waste is shown in Table 1. It did not exceed the yield strength. It is evident from the behavior that the recycled LDPE from post-consumer waste has a significantly lower breaking displacement. Further, the ultimate tensile strength for virgin LDPE was 10.36 ± 0.24 MPa, and that of recycled LDPE from post-consumer waste was 13.42 ± 0.54 MPa. These observations imply a reorganization of polymer chains in the recycled material, which is responsible for improved mechanical performance. On the other hand, virgin LDPE shows a typical regular behavior based on its uniform composition. The initial modulus of the recycled LDPE is significantly higher than virgin LDPE, as can be observed in the force-displacement curves. This is indicative of molecular reorientation during the recycling steps, which significantly improves its yield strength as well as ultimate tensile strength as compared to the virigin polymer.

The ultimate (static) tensile strength values were taken as a reference to compare the effect of cyclic loading on the tensile strength. The results show a significant difference in the achieved ultimate tensile strength. There was an approximate 30% increase in the static tensile strength of the recycled material as compared to virgin LDPE. In terms of statistical testing, recycling had a significant effect on static tensile strength (*p* < 0.05).

**Figure 5:**
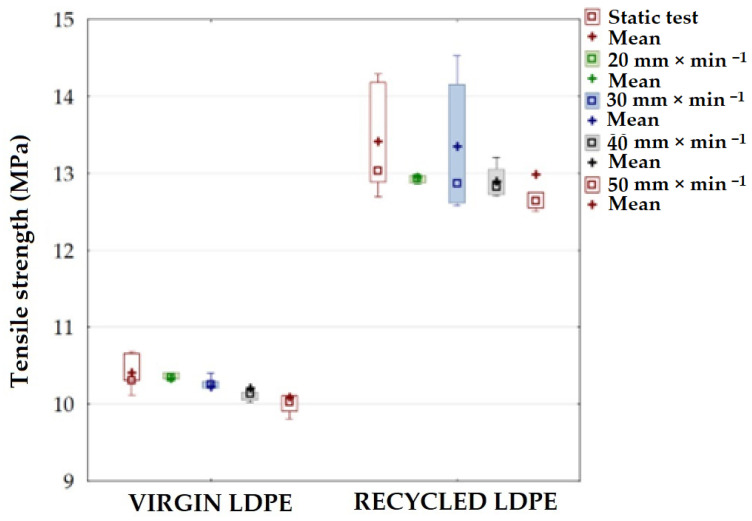
Ultimate tensile strength for virgin and recycled LDPE from post-consumer waste at different test speeds.

The cyclic test of virgin material showed a similar trend, as shown in Figure 5. The tested LDPE virgin material shows a smaller dispersion of results than the recycled LDPE material. A decrease in tensile strength with increasing test speeds of 20 to 50 mm × mm^−1^ is also evident. These findings are also supported by previous research [38]. Recycled LDPE regranulates are characterized by their high quality mainly due to several successive processing steps and the removal of impurities.

The graphical results presented in Figure 5 show the effect of test speed on the strength of LDPE material. The difference between the static tensile test results for both types of material (virgin and recycled LDPE from post-consumer waste) is significant.

The recycled LDPE material exhibited a tensile strength of 13.42 ± 0.54 MPa after static tensile testing. Similar results were achieved in the research of Moreno and Saron [38]. The cyclic loading of recycled material at a test speed of 20 mm × min^−1^ resulted in a strength of 12.95 ± 0.09 MPa; at 30 mm × min^−1^ the strength was 13.14 ± 0.77 MPa; at 40 mm × min^−1^ the strength was 12.93 ± 0.18 MPa; at 50 mm × min^−1^ the strength was 13.10 ± 0.76 MPa. Thus, there is a slight variation in strength due to the test speed in the cyclic test of recycled LDPE material relative to the static test. But this is not significant from the results presented in Figure 5, even considering the variance of the results. The difference was most significant at test speeds of 30 mm × mm^−1^ (5.89%) and 50 mm × min^−1^ (5.82%). This decrease ranged from 2.11% at a test speed of 30 mm × min^−1^ to 3.66% at a test speed of 40 mm × min^−1^. Statistical evaluation showed a significant effect of a test speed of 40 mm × min^−1^ (*p* = 0.0484). Thus, in general, the test speed had a significant effect on the resulting strength of the recycled LDPE from post-comsumer waste in fatigue cyclic tests conducted up to 80% of the maximum loading force and at different test speeds.

**Figure 6:**
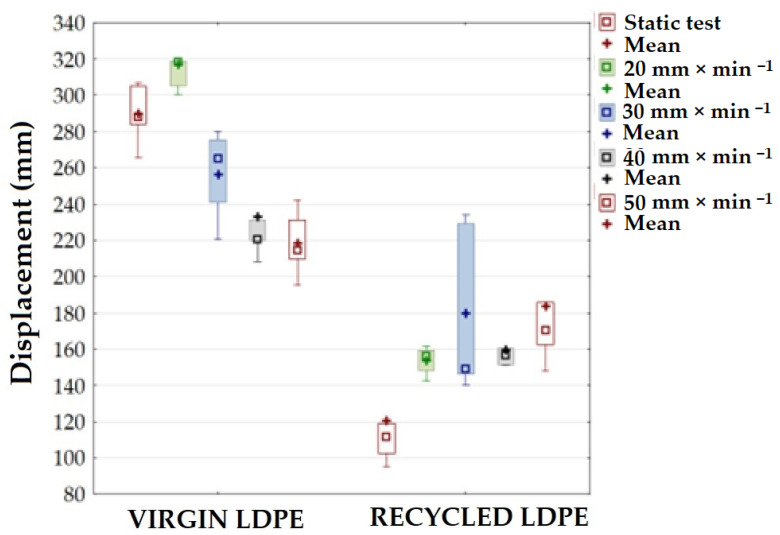
Displacement for virgin and recycled LDPE from post-consumer waste at different test speeds.

Figure 6 shows the static breaking displacement/elongation of 286.83 ± 15.46 mm for the virgin LDPE material and 126.93 ± 38.40 mm for the recycled LDPE from post-consumer waste. For the recycled LDPE material based on post-consumer waste, there was a significant reduction in displacement by approximately 56%. In statistical terms, recycling had a significant effect on breaking displacement/elongation (*p* < 0.05).

Figure 6 shows a different trend during cyclic tests in the displacement of virgin and recycled LDPE material. During cyclic tests of virgin LDPE with increasing test speeds from 20 to 50 mm × mm^−1^, breaking displacement significantly decreased.

Figure 5 shows the dependence of displacement/elongation on the test speed. After the static tensile test, the recycled LDPE material showed a displacement of 126.93 ± 38.40 mm. The displacement/elongation value of recycled LDPE material increased with increasing test speed under cyclic tensile testing. At a test speed of 20 mm × min^−1^, the value for recycled LDPE material was 153.65 ± 7.19 mm, 171.51 ± 38.55 mm at 30 mm × min^−1^, 162.30 ± 20.66 mm at 40 mm × min^−1^ and 181.52 ± 33.63 mm at 50 mm × min^−1^. The increase in displacement ranged from 21 to 43%. In terms of statistical analysis, it was shown that the lower test speed of 20 mm × min^−1^ did not significantly affect displacement/elongation compared to the static test (*p* = 0.1747). At higher test speeds, this effect was statistically significant, i.e., higher test speeds significantly affected the displacement of the recycled LDPE material (for 30 mm × min^−1^, *p* = 0.0001; for 40 mm × min^−1^, *p* = 0.0001; and for 50 mm × min^−1^, *p* = 0.0001). This increase was probably due to the fact that the relaxation capacity of the recycled LDPE material decreases as the test speed increases.

**Figure 7:**
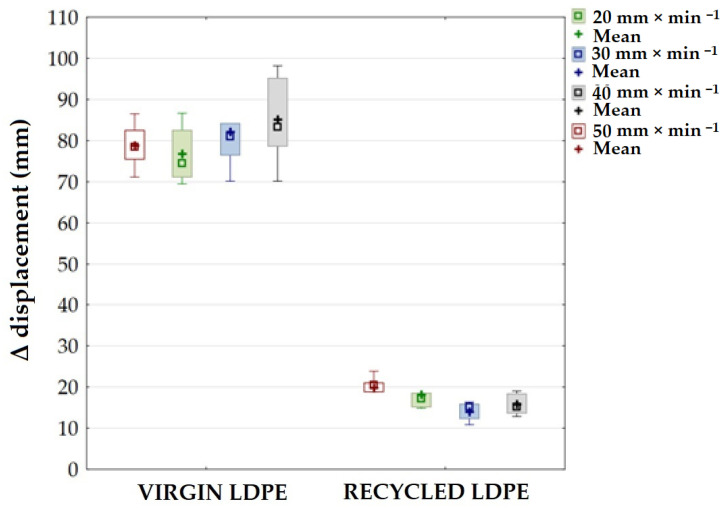
Δ displacement for virgin and recycled LDPE from post-consumer waste at different test speeds.

Figure 7 presents the difference in displacement between the 1^st^ and 1000^th^ cycles (Δ displacement), which showed cyclic creep, i.e., a quasi-static effect manifested by plastic deformation with displacement of hysteretic loops. In Figure 7, there is a noticeable difference in the resulting values of Δ displacement for the tested virgin and recycled LDPE materials. Δ displacement also varied at different test speeds by 74.8 to 83.6%. For the tested virgin LDPE material, an increasing trend in Δ displacement is evident. The results of recycled LDPE from post-consumer waste show a decreasing trend of Δ displacement with increasing test speed. Δ displacement reached a value of 19.86 ± 2.92 mm at a test speed of 20 mm × min^−1^. The value of Δ displacement for recycled LDPE material decreased with increasing test speed. It decreased to 18.47 ± 3.28 mm at a test speed of 30 mm × min^−1^ to 13.47 ± 2.22 mm at a test speed of 40 mm × min^−1^, and to 14.63 ± 2.90 mm at 50 mm × min^−1^. The results of recycled LDPE material show a significantly decreasing trend of Δ displacement with increasing test speed. Thus, it is evident that the visco-elastic behavior of recycled LDPE from post-consumer waste becomes less pronounced with increasing test speed. Due to the cyclic loading, a complex stress state was created in the material. This condition is mainly caused by the tensile stress to which the test specimens are subjected.

For products that are expected to operate in a high-cycle fatigue regime, the stress-strain states in the material are usually elastic. A longer service life is expected if the cyclic stress levels are below the yield strength of the materials [29]. For recycled LDPE-based products, a relatively shorter service life can be expected, and for this reason low-cycle fatigue testing is sufficient.

**Figure 8:**
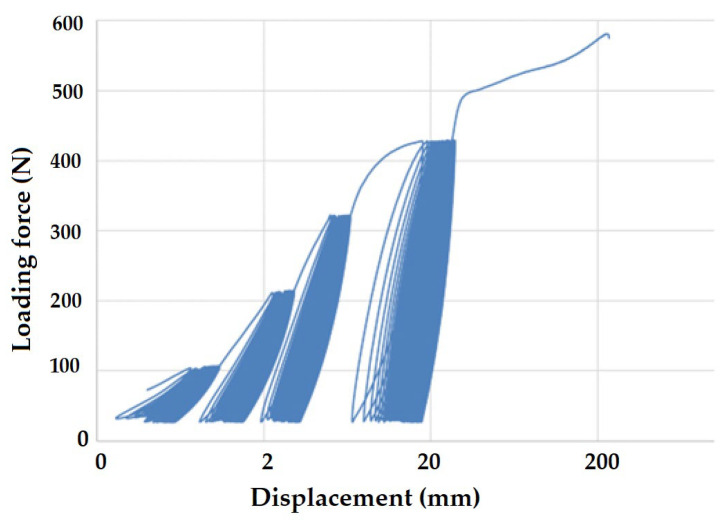
Quasi-static curve of LDPE material for a test speed of 20 mm × min^−1^ (recycled LDPE from post-consumer waste).

**Figure 9:**
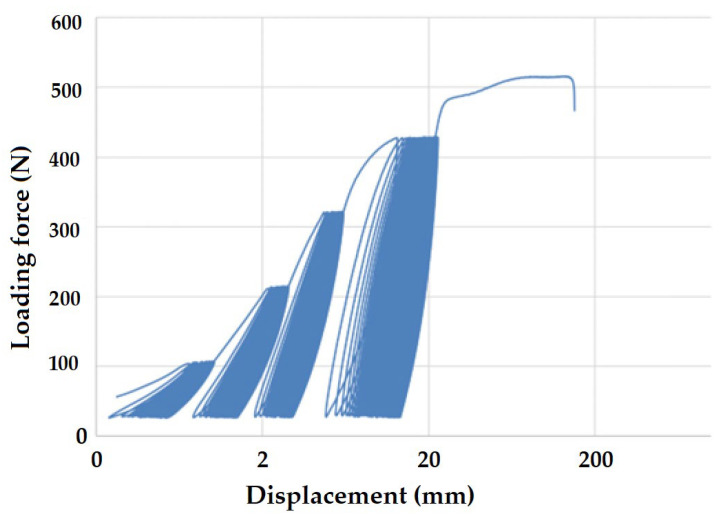
Quasi-static curve of LDPE material for a test speed of 40 mm × min^−1^ (recycled LDPE from post-consumer waste).

**Figure 10:**
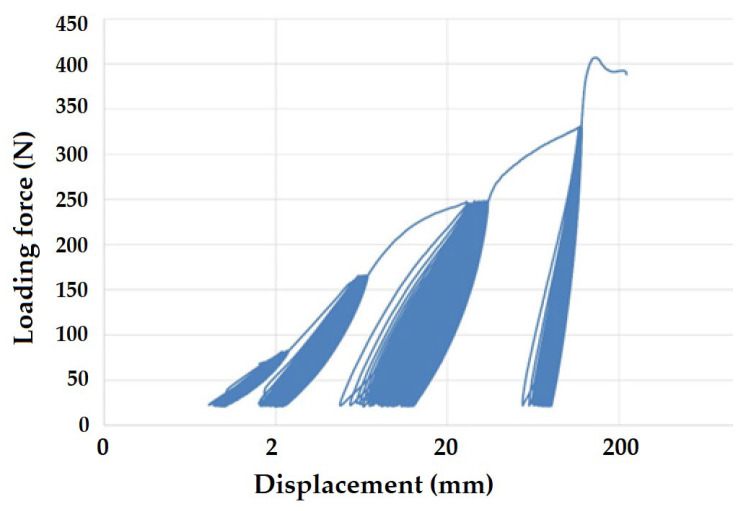
Quasi-static curve of LDPE material for a test speed of 20 mm × min^−1^ (virgin LDPE).

**Figure 11:**
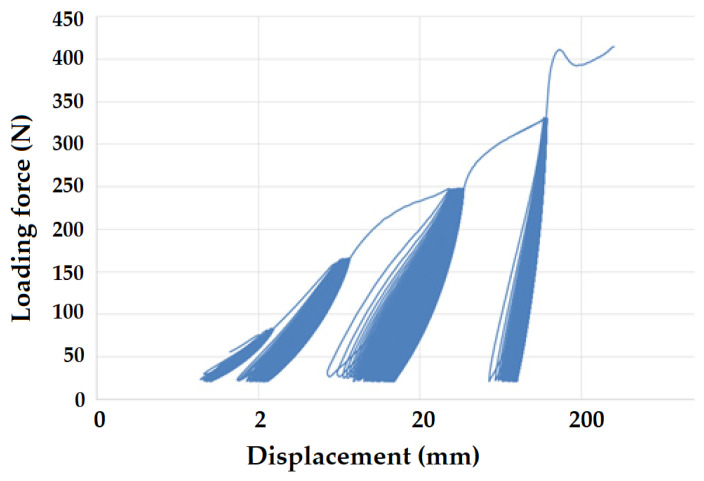
Quasi-static curve of LDPE material for a test speed of 40 mm × min^−1^ (virgin LDPE).

Figure 8 and Figure 9 present examples of quasi-static curves and hysteretic loops of recycled LDPE from post-consumer waste at two different rates of testing, respectively. To compare the results of recycled and virgin LDPE, Figure 10 and Figure 11 are presented, which show examples of quasi-static curves and hysteretic loops of virgin LDPE at two different testing speeds, respectively. From Figure 8, Figure 9, Figure 10 and Figure 11, it can be seen that 1000 cycles divided into 4 stages were first performed (see Figure 3) and then immediately loaded to failure without removing the test specimen from the universal testing machine. The tested samples showed cyclic creep, during which there is an accumulation of plastic deformation resulting from the cyclic mechanical stress manifested by plastic deformation. It is characterized by the displacement of hysteresis loops [43], i.e., the difference between the 1^st^ and the 1000^th^ cycles. This causes permanent deformation due to the fatigue behavior of the injection-molded recycled LDPE from post-consumer waste. The permanent deformation after the last 1000 cycles at different test speeds can be seen from Figure 7. A decreasing trend of this permanent deformation with increasing test speed was observed. Test speed is another important factor that affects the permanent deformation. Previous research has shown that permanent deformation increases with increasing speed in cyclic tensile testing [44,45]. This assumption was also supported by the virgin LDPE tested.

During fatigue testing, i.e., cyclic loading, there was no damage to the recycled or virgin materials tested. This assumption is essential for its practical application and gives a prerequisite for a successful recycling process in practice. In fatigue tests, unforeseen failures of material integrity often occur due to the accumulation of plastic deformation. Plastic deformation is the cause of fatigue in recycled LDPE [46,47].

Figure 8 presents the curve for a test speed of 20 mm × min^−1^ cyclic loading, and Figure 9 presents the curve for a test speed of 40 mm × min^−1^ for recycled LDPE from post-consumer waste. The curves show the described Δ displacement at different test speeds. Similar behavior can be seen in the tested virgin LDPE from Figure 10 and Figure 11.

For polymeric materials, the response to cyclic loading is primarily viscoelastic, but in the case of higher values of cyclic loading, cracking may also occur [48]. The viscoelastic response to cyclic loading is given mainly by the medium level stress and its amplitude [48]. Therefore, cracking is one of the key factors in the design of structural elements, which should be considered when using polymeric materials such as LDPE. A better understanding of fatigue behavior is essential for the application of recycled polymeric materials in practice [49,50,51].

The quasi-static curves show a similar shape and drift of the hysteresis loops. This condition was described during research on PA6.6 composites [29,49]. It can also be stated that the course was not stabilized, and the shape and size changed with the increasing number of cycles [29,52]. An accumulation of progressive mean strain per cycle took place due to the drift in the hysteresis loops [53]. Figure 8, Figure 9, Figure 10 and Figure 11 show the different slope of the hysteresis loops when the loading force changes in the progressive cyclic test. A significant difference in the slope of the hysteresis loops is noticeable, especially at lower loading forces.

**Figure 12:**
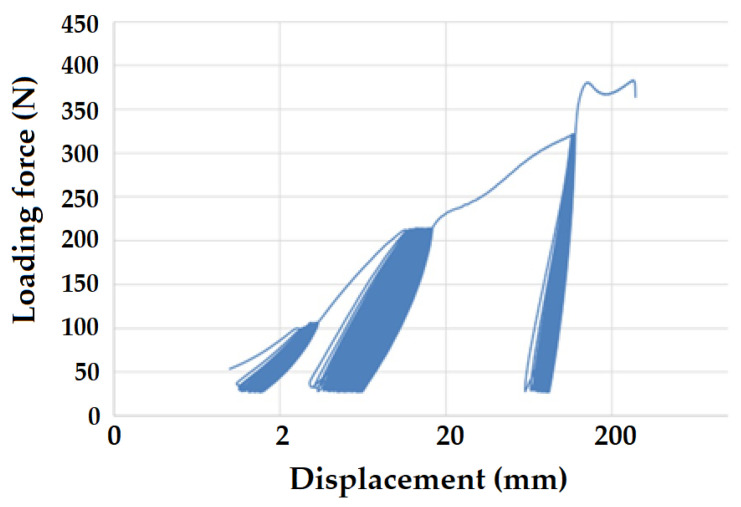
Quasi-static curve of virgin LDPE material for a test speed of 50 mm × min^−1^—setting the amplitude of the sequential cyclic test according to recycled LDPE from post-consumer waste.

Figure 12 shows an example of the quasi-static curve of the hysteretic loops of the tested virgin LDPE material at a test speed of 50 mm × min^−1^ and the test settings according to recycled LDPE from post-consumer waste listed in Table 1. The loading force values during individual cycles based on the static tensile test were therefore greater compared to the virgin material. The test ended at the transition between the third and fourth testing amplitudes, i.e., between 322 N and 429 N. The value for the static tensile strength of the virgin LDPE material was 414.4 ± 21.6 N. The last 250 test cycles at a load of 27 to 429 N were not started due to premature destruction of the tested material. Destruction occurred at 379.8 ± 8.26 N (i.e., 10 MPa). The upper limit of amplitude was therefore up to 92%, which was 12% compared to the setting based on the reference value of the force during the static test of virgin LDPE. It was clear from the results that virgin LDPE exhibited a lower static and cyclic tensile strength than the recycled LDPE from post-consumer waste. This test example shows that it is not possible to subject virgin LDPE to higher values of cyclic loading. During fatigue tests, i.e., cyclic loading, the tested recycled materials were damaged during the test. In this test, the cyclic creep or Δ displacement was 84.31 ± 11.5 mm. A similar value was achieved in the comparative tests shown in Figure 7.

When comparing the test specimens visually during loading, a significant secondary neck was evident in the virgin LDPE, where the ultimate failure occurred.

**Figure 13:**
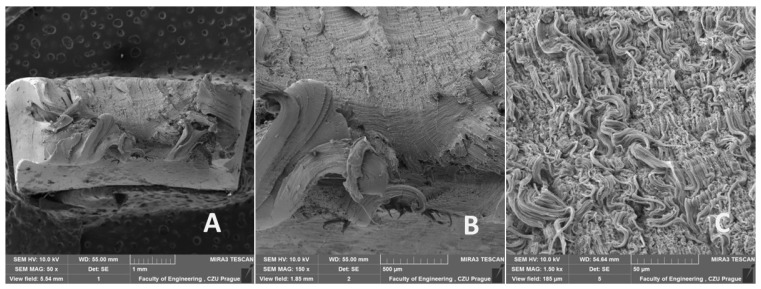
SEM images of the fracture surface after static tensile testing of recycled LDPE from post-consumer waste: (**A**): MAG 50×, (**B**): MAG 150×, (**C**): MAG 1.50 k×.

**Figure 14:**
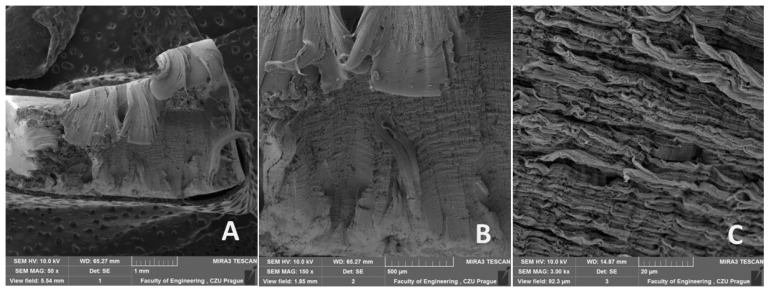
SEM images of the fracture surface after fatigue cyclic test at test speed 30 mm × min^−1^ of recycled LDPE from post-consumer waste: (**A**): MAG 50×, (**B**): MAG 150×, (**C**): MAG 3.00 k×.

**Figure 15:**
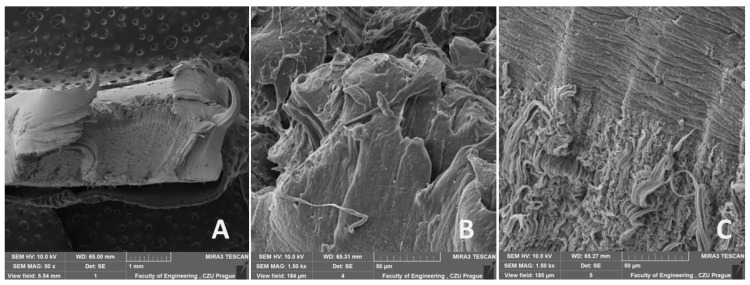
SEM images of the fracture surface after fatigue cyclic test at test speed 50 mm × min^−1^ of recycled LDPE from post-consumer waste: (**A**): MAG 50×, (**B**): MAG 150×, (**C**): MAG 1.50 k×.

The morphology of the fracture surfaces of recycled LDPE from post-consumer waste is shown in Figure 13, Figure 14 and Figure 15. The semi-crystalline thermoplastic polymers exhibit peculiar behavior in static uniaxial tensile tests, evident from Figure 13. Similar behavior is also visible in fatigue cyclic tests at different loads and test speeds, as shown in Figure 14 and Figure 15. After the initial yield strength limit was reached, a local narrowing of the test specimen and neck propagation occurred. This is caused by the crystalline blocks and chains oriented in the direction of the loading force and can be seen from Figure 13A, Figure 14A and Figure 15A. LDPE material is characterized by high ductility and stretches to form a distinct neck throughout the cross section. At the moment of breakage, the polymers again shrink in the opposite direction, which is shown in Figure 13A,B, Figure 14A,B and Figure 15A,B. A more detailed view of the breakage can be seen in Figure 13B, Figure 14B and Figure 15B. From Figure 13C, Figure 14C and Figure 15C, the homogeneous structures can be seen without large pores. It is due to the high pressure and temperature during the plasticization phase of the melt during the production of the test samples from the granulate by the injection molding process. There was no difference in the damage mechanism between the static tensile test and different cyclic fatigue tests. From Figure 13C, Figure 14C and Figure 15C, the parallel polymer chains can be seen in the direction of loading.

A smooth, non-porous structure with no obvious defects was achieved by injection molding by regranulation of LDPE from post-consumer waste. The recycling process did not cause internal defects using the injection molding process, which is visible from Figure 13, Figure 14 and Figure 15 (SEM images). The SEM images show a homogeneous structure with parallel lines oriented in the loading direction and wavy areas in the transverse direction caused by shrinkage during failure/rupture.

### 3.2. FTIR Analysis

**Figure 16:**
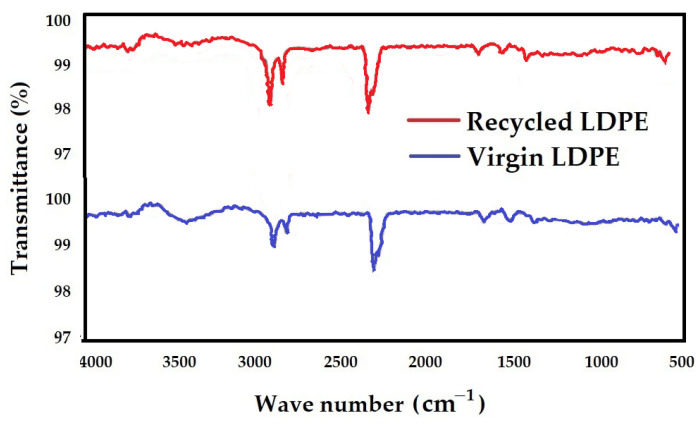
FTIR of virgin and recycled LDPE from post-consumer waste.

In infrared spectroscopy, IR radiation is passed through the samples. Some of the infrared radiation is absorbed by the sample, and some of it is transmitted. The resulting spectrum represents the molecular absorption and transmission, creating a molecular fingerprint of the sample. Like a fingerprint, no two unique molecular structures produce the same infrared spectrum. This makes infrared spectroscopy useful for several types of analysis. The transmittance results for virgin LDPE and recycled LDPE from post-consumer waste are presented in Figure 16.

It can be observed that the transmittance patterns of both virgin and recycled LDPE from post-consumer waste are very similar. It is indicative of similar compositions and functional groups in both types of samples. The % composition of the functional groups is determined by a normalization procedure. Here, the peaks are integrated and allocated to possible functional groups. The functional group assigned to a specific wave number band is based on instances in the literature [54,55,56,57,58,59].

The various functional groups and their approximate % composition in the virgin and recycled LDPE from post-consumer waste are given in Table 3.

The virgin and recycled LDPE from post-consumer waste show similar functional groups and compositions. The unknown other substances are slightly reduced in the regranulates of the recycled LDPE from post-consumer waste. This supports the results of mechanical analysis, which shows an increase in the static and cyclic tensile strength of the recycled LDPE from post-consumer waste as compared to the virgin material.

### 3.3. Differential Scanning Calorimetry (DSC)

**Figure 17:**
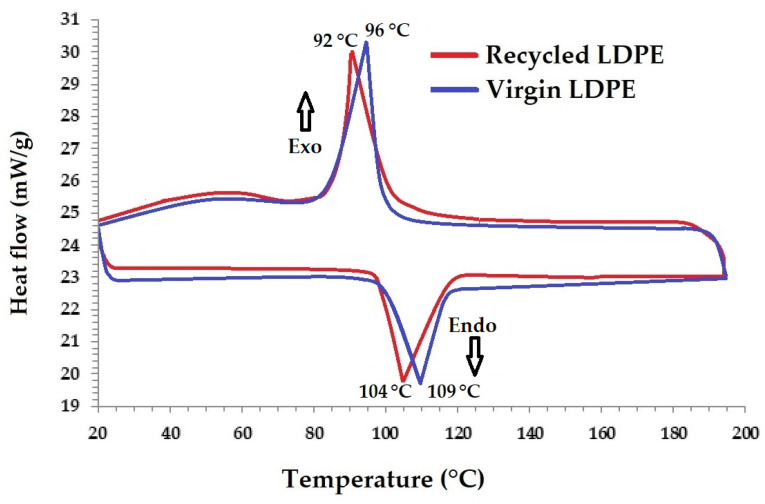
DSC thermogram of virgin and recycled LDPE from post-consumer waste.

The thermal properties of polymers also indicate the physical response to the application of heat and the resultant change in temperature in response to the applied heat. The physical impact of temperature on materials is reversible in cases of short-term application, whereas it is nonreversible in long-term application as it associates with the internal chemical changes. The long-term effects at elevated temperatures are aging/degradation, resulting in variations in the mechanical, physical and chemical properties. The thermal behavior of virgin LDPE and recycled LDPE from post-consumer waste was analyzed using a DSC thermogram, as shown in Figure 17.

The DSC thermogram shows the heat flow from molecular transitions as a function of time and temperature. This provides both qualitative and quantitative information on physical and chemical changes because of endothermic (heat absorption) and exothermic (heat release) processes, respectively. The peaks of the melting (endothermic) and crystallization (exothermic) phases reflect the thermal change of LDPE samples at the molecular level. Figure 17 shows the DSC thermograms of the virgin and recycled LDPE from post-consumer waste that were studied. The endothermic and exothermic events are more prevalent than the others.

It was observed that the endothermic peak emerges in both virgin and recycled LDPE from post-consumer waste around a similar temperature range as it increases from room temperature to 100–110 °C. It is linked to the melting of the crystal structures of polymers. There is a small difference in melting points between the samples tested. Recycled LDPE from post-consumer waste shows a slightly lower melting point (peak temperature) as compared to virgin polymer. The crystallinity was measured during the second heating cycle in the DSC procedure. A difference in the peak can be attributed to a slightly higher crystallinity in the virgin LDPE as compared to recycled LDPE from post-consumer waste. Exothermic peaks were also seen at about 90–100 °C. This was observed for both virgin and recycled samples around the same temperature range. As compared to the virgin LDPE, the recycled samples show a slightly lower thermal peak, indicating a slightly lower thermal stability. It might be due to a slightly lower crystallinity (33.5%) in the case of recycled LDPE from post-consumer waste as compared to 33.9% in the virgin LDPE sample. However, the difference in crystallinity was not very significant. The results of DSC indicated that there was no major degradation of the recycled LDPE from post-consumer waste as compared to virgin LDPE.

### 3.4. Dynamic Mechanical Analysis (DMA)

**Figure 18:**
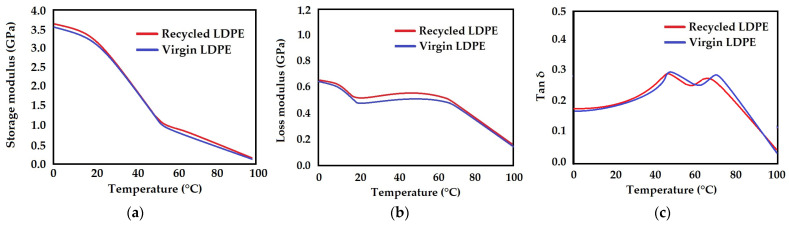
DMA thermograms of virgin and recycled LDPE from post-consumer waste; (**a**) Storage modulus, (**b**) Loss modulus, (**c**) Tan δ.

The DMA thermograms of virgin and recycled LDPE from post-consumer waste are described as a function of temperature in Figure 18a–c.

The DMA thermograms usually indicate the β- and α- transitions corresponding to the glass transition and the melting of the thinnest lamellae, respectively. As seen from Figure 18a, there is a decreasing trend in the storage modulus (decrease in stiffness) over the entire range of experimental temperatures. Thermoplastic polymers such as LDPE are semi-crystalline, so they have a high degree of orientation, which strongly affects their mechanical properties. At lower temperatures, the molecules are so immobile that they are unable to resonate with the oscillatory loads and therefore remain stiff. The macromolecular segments cannot change shape, especially through rotation about C–C bonds, and so the molecular entanglements act as rigid crosslinks. At elevated temperatures, the molecular segments become mobile and have no difficulty resonating with the load. When the timescale of molecular motion coincides with that of mechanical deformation, each oscillation is converted into the maximum-possible internal friction and non-elastic deformation. The entanglements remain firmly in place but may occasionally slip and become disentangled. The loss modulus, which is a measure of this dissipated energy, also reaches a maximum and then decreases. It is important to mention that the modulus is determined primarily by the strength of the intermolecular forces and the way the polymer chains are packed. At higher temperatures, mobility increases, and the molecules lose their packing arrangement.

The loss tangent, or tan δ, is a ratio of the loss modulus to the storage modulus and is measured as the mechanical loss or damping factor. The damping properties of the material act to balance between the elastic and viscous phases in a polymeric structure. In nature, tan δ is the response of inner frictional forces. Figure 18c presents the Tan δ curves of the virgin and recycled LDPE from post-consumer waste. In addition to the α- and β- transitions, LDPE shows two transition peaks that may be called the α- and α*- transitions. The α- transition is related to larger chain segments in the amorphous phase that start to move, while the α*- transition is associated with the slippage between crystallites [60,61,62,63]. Another explanation for the double transition may be that there is melting of the smallest crystallites closely followed by interlamellar shear of the larger crystallites.

The variations of tan δ in virgin and recycled LDPE are very similar. It can be observed that there was an increasing trend in the loss tangent up to 50 °C. This can be justified by the restriction of the motion of polymer chains. Subsequently, there is a small decrease to around 60 °C, before further increasing to 65–75 °C. Then, it continuously decreases to 100 °C. The behavior of virgin LDPE and recycled LDPE is quite similar. The α- transition appears at a slightly higher temperature for the virgin LDPE as compared to the recycled LDPE from post-consumer waste, which may be the result of the immobilization of the polymer chains owing to slightly higher crystallinity. The shift in curves with the second peak may be attributed to the molecular reorganization in the recycled LDPE during the processing steps rather than any change in the virgin material itself. These possible variations have also been reported in the literature [60,61,62]. These changes might be due to the processing during recycling [60,61]. These changes might have also influenced the improvement in mechanical performance in recycled LDPE from post-consumer waste, as reported earlier. These observations are in line with the findings of mechanical performance in recycled LDPE from post-consumer waste, which do not exhibit any significant changes after 1000 cycles of tensile loading. Rather, the static tensile strength of the recycled LDPE from post-consumer waste was higher than the virgin material.

Though the recycled LDPE material performed quite satisfactorily with respect to mechanical and thermo-mechanical performance, it is difficult to use such recycled plastics from post-consumer waste for more demanding applications, such as long-term packaging. These materials do not meet industry requirements for color, odor or the migration of unknown organic substances, etc. The smell of the tested materials was also not proven.

## 4. Conclusions

In this paper, results are published on the issue of fatigue life under cyclic loading for recycled LDPE from post-consumer waste. Cyclic fatigue reduces the life cycle of recycled polymer products, which can be degraded by use or improper manufacturing techniques. The results of the current research carried out on fatigue tests running up to 80% of the maximum force under a static tensile test proved the functionality of the tested material produced by the injection molding process. Even though it was recycled post-consumer waste, the material showed a very good service life. The advantages of LDPE are that it is non-toxic and non-hazardous to health, while at the same time it is tasteless, odorless and does not absorb water. This fact is essential for the recycling of post-consumer waste material.

Based on the results presented in this article, the following conclusions can be drawn:

- The injection molding method achieved a smooth, non-porous structure with no obvious defects in the recycled LDPE from post-consumer waste material.
- Cyclic loading is one of the most common causes of the premature failure of polymeric materials. While using recycled material based on post-consumer wastes, the risk of violation is even more significant due to degradation processes. Research has shown that the recycled LDPE from post-consumer waste does not show deterioration in the mechanical or thermo-mechanical properties or their premature loss during the cyclic loading test. It was found that the mechanical properties of the recycled LDPE from post-consumer waste produced by the injection molding method are similar or even better than the original virgin material. The tested material did not undergo destruction due to cyclic fatigue tests taking place up to 80% of the maximum force during static testing.
- No decrease in tensile strength was observed for the recycled LDPE from the post-consumer waste material tested. No significant effect of the test speed was shown on the results of the static tensile test and the fatigue cyclic tests running up to 80% of the maximum load. Displacement increased significantly due to cyclic fatigue tests compared to static tensile test results. The cyclic fatigue tests at different test speeds resulted in permanent deformation between the 1st and 1000th cycles due to the fatigue behavior of the injection-molded recycled LDPE from post-consumer waste.
- Using SEM analysis, the fracture surface of the injection-molded recycled LDPE from post-consumer waste material was observed, which showed a homogeneous structure with parallel lines oriented in the loading direction and wavy regions in the transverse direction resulting from the shrinkage of the broken polymer chains.
- The FTIR spectra obtained for both virgin and recycled LDPE from post-consumer waste were quite similar. Further, the melting (endothermic) and crystallization (exothermic) peaks observed by DSC thermogram were similar for virgin and recycled LDPE from post-consumer waste. DMA thermograms showed similar results for the storage modulus, loss modulus and loss tangent (tan δ) for both virgin and recycled LDPE from post-consumer waste. The recycling did not cause much thermal damage to LDPE obtained from post-consumer waste.

The obtained results are promising in terms of providing a sustainable and environmentally friendly approach to recycling and reusing LDPE waste materials to develop value-added products with sufficient mechanical and thermomechanical performance. Such recycled LDPE can be used for several other lightweight home appliances, fittings, etc., apart from packaging applications. In future research, several other mechanical properties of the recycled LDPE-based material, e.g., flexural rigidity, hardness, shear modulus, etc., can be evaluated for a more comprehensive understanding of the performance as compared to virgin LDPE. Further, its electrical, thermal and other properties can be studied. Further research can be conducted to study the durability and serviceability of products developed from such recycled material.

## Figures and Tables

**Table 1:** Setting the amplitude of the sequential cyclic test for one test of recycled LDPE from post-consumer waste.

Lower Amplitude Limit	Upper Limit of Amplitude	Number of Cycles within the Amplitude
27 N	107 N	250
27 N	215 N	250
27 N	322 N	250
27 N	429 N	250

**Table 2:** Setting the amplitude of the sequential cyclic test for one test of virgin materials.

Lower Amplitude Limit	Upper Limit of Amplitude	Number of Cycles within the Amplitude
21 N	83 N	250
21 N	166 N	250
21 N	248 N	250
21 N	331 N	250

**Table 3:** Various functional groups and their approximate % in virgin and recycled LDPE from post-consumer waste.

Functional Group	Wave Number Band	% in Virgin LDPE	% in Recycled LDPE from Post-Consumer Waste
(=CH−)	2900–4000	34.4	34.6
(−CH_3_)	2840–2860	22.2	22.7
(C-C)	2340–2370	8.7	8.4
(C=O)	1810–1820	0.7	0.6
(C=C)	1640–1650	1.5	1.2
(−COOH)	1370–1380	2.6	3.4
(−OH)	3250–3580	4.4	4.8
(=CH_2_)	1450–1470	16.9	16.5
Others	710–910	8.6	7.8

## Data Availability

The original contributions presented in the study are included in the article, further inquiries can be directed to the corresponding author/s.
